# The Pulse-Respiration Quotient: A Powerful but Untapped Parameter for Modern Studies About Human Physiology and Pathophysiology

**DOI:** 10.3389/fphys.2019.00371

**Published:** 2019-04-09

**Authors:** Felix Scholkmann, Ursula Wolf

**Affiliations:** Institute of Complementary and Integrative Medicine, University of Bern, Bern, Switzerland

**Keywords:** pulse-respiration quotient, PRQ, cardio-respiratory coupling, integrative human physiology, chronobiology

## Abstract

A specific and unique aspect of cardiorespiratory activity can be captured by dividing the heart rate (HR) by the respiration rate (RR), giving the *pulse-respiration quotient* (PRQ = HR/RR). In this review article, we summarize the main findings of studies using and investigating the PRQ. We describe why the PRQ is a powerful parameter that captures complex regulatory states of the cardiorespiratory system, and we highlight the need to re-introduce the use of this parameter into modern studies about human physiology and pathophysiology. In particular, we show that the PRQ (i) changes during human development, (ii) is time-dependent (ultradian, circadian, and infradian rhythms), (iii) shows specific patterns during sleep, (iv) changes with physical activity and body posture, (v) is linked with psychophysical and cognitive activity, (vi) is sex-dependent, and (vii) is determined by the individual physiological constitution. Furthermore, we discuss the medical aspects of the PRQ in terms of applications for disease classification and monitoring. Finally, we explain why there should be a revival in the use of the PRQ for basic research about human physiology and for applications in medicine, and we give recommendations for the use of the PRQ in studies and medical applications.

## Introduction

Cardiac activity and respiration can been seen as constituting a complex non-linear dynamical system based on two weakly coupled oscillators (heart beating and breathing movement) coupled by several structural and functional types of *cardiorespiratory interaction* (CRI) These CRI types involve complex interplay between the activity of the brainstem (with the autonomic nervous system and the central respiratory drive as main elements), the heart and lungs in the thoracic cavity, and the vascular system (Valenza et al., 2016; Benarroch, 2018; Elstad et al., 2018). The CRI gives rise to several types of emergent *cardiorespiratory coupling* (CRC) phenomena characterized by specific relationships between cardiac activity and respiration with respect to frequency, phase, and time differences between the heart beats and the respiration events (Lotrič and Stefanovska, 2000; Moser et al., 2008; Schulz et al., 2013; Dick et al., 2014; Krause et al., 2017; Elstad et al., 2018).

One specific type of CRC is the relationship between the heart rate (HR), colloquially the “pulse,” and the respiration rate (RR). This relationship can be analyzed in the framework of the cardiorespiratory synchrogram (i.e., analyzing the relative phases of the heart rate and respiration rate as a function of time (Schäfer et al., 1998) or simply as the ratio of the HR to the RR, termed the *pulse-respiration quotient* (PRQ = HR/RR).^1^

The aim of this review article is to summarize the main findings and studies using and investigating the PRQ. In addition, we describe why the PRQ is a powerful parameter that captures complex regulatory states of the cardiorespiratory system, and we highlight the need to re-introduce the use of this parameter into modern studies about human physiology and pathophysiology.

The PRQ was used in many studies in the second half of the last century, mainly in papers published in German scientific journals. This explains why the many findings about the PRQ are currently not appreciated by the non-German-speaking scientific community. With this review article, we want to make the knowledge also available to the English-speaking scientific community, and draw a special attention to the fact that the PRQ has great potential for use in future studies to give additional and novel insights into the functioning of the human body.

## Essentials About the PRQ

### The PRQ: Definition and Terminology

The PRQ can be used as the ratio of the *instantaneous* HR to the *instantaneous* RR, or as the ratio of the HR to the RR measured in a defined *time interval*, e.g., over 1 min.

For a clear discussion of the published findings regarding the PRQ and its relationship to human physiology and pathophysiology, the following new terminology is introduced that distinguishes three basic types of PRQ parameters:

- rs-PRQ: *resting-state* PRQ, i.e., the PRQ measured during a resting state of the body. Normally, a specific protocol is used for standardized testing, e.g., the PRQ measured in the supine body posture of the first minute after 10 min of resting to establish a baseline state. If a person is sleeping (during the day or night), then the PRQ is automatically a rs-PRQ. We regard the supine body posture as strictly required in order to determine the rs-PRQ. It is well known that the body posture has a significant impact on the cardiorespiratory state (Iida et al., 1999; Gordon et al., 2011; Silvani et al., 2017).
- f-PRQ: *functional* PRQ, i.e., the PRQ measured during a functional task that the subject has to perform or a specific stimulation the subject is faced with. This enables the regulatory capacity of human physiology to be determined.
- u-PRQ: *unrestricted* PRQ, i.e., the PRQ measured during normal daily activities without changing the cardiovascular system especially for the PRQ measurements. This type of PRQ is normally measured when performing 24 h PRQ measurements.

Furthermore, it is necessary to distinguish when the PRQ was determined, during *night* or *day*. This can be indicated by adding a “D” (for day, i.e., the time between sunrise and sunset) or an “N” (for night, i.e., the time between sunset and sunrise) in front of the term, leading to 2 × 3 PRQ sub-classes (for a visualization, see Figure 4G), i.e., D-rs-PRQ, D-u-PRQ, D-f-PRQ, N-rs-PRQ, N-u-PRQ, and N-f-PRQ. Sub-classes of PRQ parameters can be also formed by employing the state (rs-, u-, f-) or the time (N-, D-) indices only, i.e., rs-PRQ or N-PRQ.

Distinguishing different types of PRQ parameters is important since the PRQ behavior is substantially determined also by the time of measurement. In many published papers, it is difficult to infer the PRQ subtype since only the umbrella term “PRQ” is used. In the following, the umbrella term “PRQ” is only used when referring generally to the PRQ.

### Historical Development, Measurement Methods, Basic Characteristics, and Link to Cardiorespiratory Interactions

The fact that the HR of humans is approximately four times faster than the RR has been known for millennia, but it was the Austrian scientist Rudolf Steiner, who emphasized the great potential of the PRQ for our understanding of human physiology. This is especially true with respect to the significance of the PRQ value of 4 (i.e., a 4:1 ratio of the cardiac activity to respiration) (Steiner, 1989). Since then, the PRQ has been mainly investigated in physiological or medical studies by German research groups (e.g., Hildebrandt, 1955, 1960; Weckenmann, 1975; Weckenmann et al., 1988; Moser et al., 2008; Von Bonin et al., 2014).

**Figure 1:**
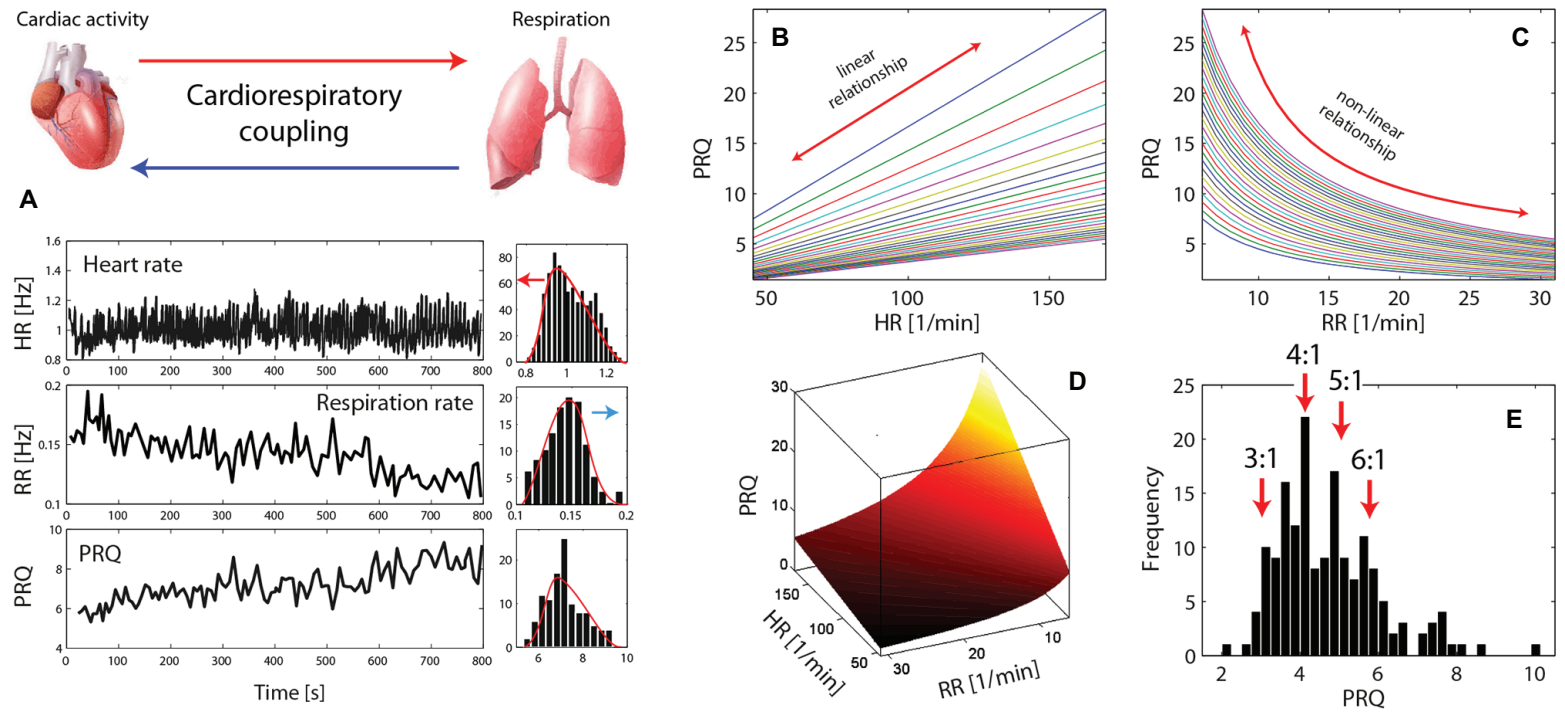
**(A)** Illustration of the principle of cardiorespiratory coupling (CRC) and exemplary time series of heart rate (HR), respiration rate (RR), and the pulse-respiration quotient (PRQ) (data extracted from Lotrič and Stefanovska, 2000). On the right side of each time series, the histograms are shown exhibiting clearly the phenomenon of the complex interplay between the intrinsic oscillations and coupling of the oscillators causing a deviation of the intrinsic frequencies as evident by the skewed distributions of HR and RR. The directions of deviations are marked with arrows. **(B–D)** The relationship of the PRQ with HR and RR [simulation; normal ranges of human HR and RR values are used (from resting state to intense physical activity), i.e., HR = 45–170 1/min, RR = 6–31 1/min]. **(D)** PRQ-HR-RR space where the extreme PRQ values (PRQ > 10 and < 2) are mainly due to pathophysiological states. **(E)** The quantization phenomenon of the PRQ (*n*:*m* ratio with *n* = 3–6 and *m* = 1 is shown as often observed in human PRQ studies) (data extracted from Moser et al., 2008).

Capturing several aspects of the CRC at once, the PRQ contains information about several specific forms of CRC, i.e., *cardiorespiratory frequency locking* (constant PRQ over a specific time interval) or *cardioventilatory coupling* (CVC) (causing the occurrence of specific PRQ values). The CRC is the result of bidirectional CRI effects that adjust the cardiac and respiratory activities due to the current physiological state and needs. Both the cardiac and respiratory activities can be regarded as linear and non-linear oscillators with *intrinsic* (autonomous) oscillations of specific frequencies that are constantly adjusted, resulting in the *actual* (measurable) frequencies. Linked to this phenomenon is the fact that the intrinsic HR is larger than the actual one (Katona and Jih, 1975), and the intrinsic RR is smaller (Karczewski and Widdicombe, 1969). The complex CRIs result in several observable phenomena (see Figure 1), e.g., the *skewed* distributions of HR and RR values at rest (due to the interplay between the intrinsic and actual frequencies), a *lognormal* distribution of the PRQ (with a higher likelihood of smaller values than larger ones), and a *quantization* of the PRQ values with preferred values of the harmonic ratios *n*:*m* with *n* = 3–6 and *m* = 1. The rs-PRQ values normally show distribution peaks at 3 and 4, corresponding to 3:1 and 4:1 HR and RR ratio values. The cardiorespiratory state where the PRQ exhibits a value around 4 was termed “*PRQ normalization”* (i.e., a state of an “optimal” PRQ with respect to the functioning of the cardiovascular system) to highlight the significance of this state for human physiology (Hildebrandt, 1960).

The PRQ can be regarded as a parameter incorporating the information about the HR and RR, while both determine the PRQ differentially (the HR in a linear way and the RR in a non-linear way). For a visualization of this aspect, see Figures 1B–D. The PRQ thus represents an emergent behavior that cannot be obtained when analyzing the HR and RR separately (as normally done in studies about human physiology and pathophysiology). As a further indication of the significance of the PRQ, it can be noted that almost all physiological variables of biological organisms (mammals) are body mass-dependent, i.e., they follow allometric scaling laws (Schmidt-Nielsen, 1984; West et al., 1999), but there are a few exceptions and the PRQ is one of them. The PRQ is independent of the mass of the organism and approximately 4.5 for *all* mammals (Stahl, 1967; Schmidt-Nielsen, 1984) – an interesting fact that should be subject to further investigations.

As a parameter that is sensitive to the current state of the human cardiorespiratory system (CRS), as well as linked to the cardiovascular system (CVS) and the autonomic nervous system (ANS), the PRQ is influenced by internal and external factors. Eight main factors have been identified: *age*, *sex*, *chronobiology*, *body posture*, *behavior*, *physiological constitution*, *environmental influences,* and the *health/disease state*.

The PRQ is related to the CRS and CVS in parallel since both the HR and the RR are a result of the structural (anatomical) and functional (physiological) state of cardiac activity and respiration, which in turn are determined and modulated by the current state of the vascular system (Elstad et al., 2018). Since the state of the CRS and CVS is influenced by various other systems’ states (e.g., immune system, endocrine system, and nervous system) and the state of the structural and functional relationships within and between these systems (as described, e.g., by chronobiology and systems medicine), the PRQ is naturally a useful parameter to capture the overall current state of human physiology in general.

### Measurement of the PRQ

**Figure 2:**
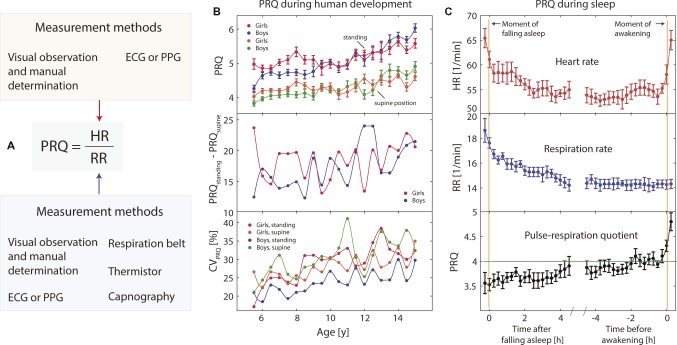
**(A)** Visualization of the main methods of measuring the pulse-respiration quotient (PRQ). **(B)** PRQ during human development [*n* = 1,820, age range: 5.5–15 years, daily resting-state PRQ (D-rs-PRQ) measurements] as a function of age, sex, and body position (data extracted from Matthiolius and Hildebrandt, 1995). CV: coefficient of variation, error bars: standard error of the mean. **(C)** Typical changes of heart rate (HR), respiration rate (RR), and PRQ during sleeping (data extracted from Pöllmann and Hildebrandt, 1970; *n* = 7). The PRQ normalization tendency during sleep (especially during the last hours before awakening) is clearly visible. Error bars: standard error of the mean.

There are different ways to measure the PRQ, depending on how the HR and RR are determined (see Figure 2A). One way is to determine the HR and RR using only one single measurement method, i.e., electrocardiography (ECG) or photoplethysmography (PPT). In this case, the HR (or the pulse rate, PR, in case of PPT) can be extracted as well as the RR. There are several algorithms available to determine the RR based on ECG or PPT data (e.g., Schäfer and Kratky, 2008; Nilsson, 2013; Charlton et al., 2018). The RR can also be measured directly using a thermistor, a respiration belt, an audio analysis, thermal imaging or by analyzing the end-tidal CO_2_ (P_ET_CO_2_) waveform obtained by capnography (Al-Khalidi et al., 2011). In several of the published PRQ studies (especially the older ones), the PRQ was simply calculated by manually determining the PR and HR (by palpation of the pulse and visual observation of the respiration-related movements of the chest). Since each HR and RR determination method has its own unique accuracy and precision, the resulting PRQ is always to some degree dependent on the methods applied. Studies performing a comparison of different methods to determine the PRQ have not been performed so far, but it has already been noted that the way the PRQ is determined can have a significant impact on the results (e.g., the calculated numerical PRQ value depends on the method used) (Heckmann, 2001). It is therefore recommended to use the most direct and up-to-date approach for measuring the HR and RR and to report precisely how the PRQ was determined when publishing a study involving the PRQ. It is expected that the precision and accuracy are not really an issue when working with averaged PRQ values over relatively long-time intervals (minutes or hours), but a precise PRQ is necessary when an accurate time resolution of the PRQ changes is relevant (often the case when determining f-PRQ values).

## PRQ in the Context of Human Physiological Development, Chronobiology, and Somnology

### The PRQ Changes During Human Development

**Figure 3:**
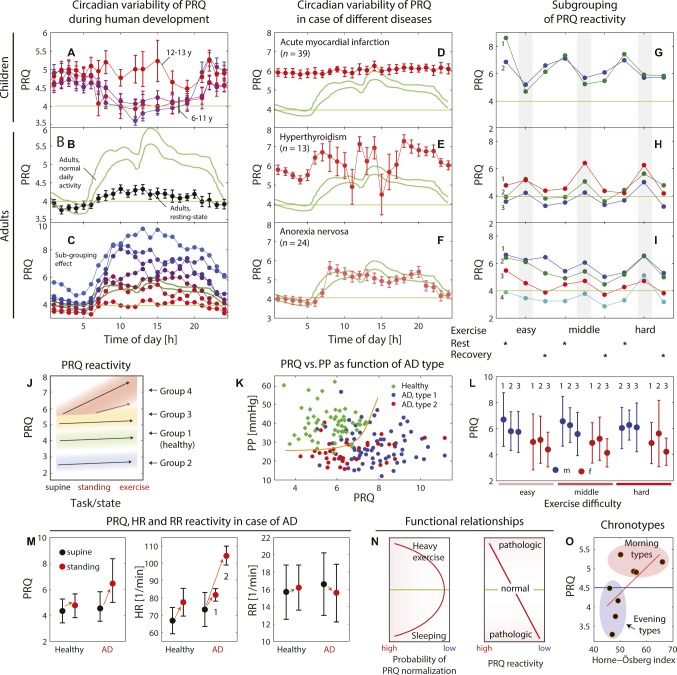
**(A–C)** Dependence of the circadian pulse-respiration quotient (PRQ) oscillation on age and measurement condition (data extracted from Breithaupt et al., 1980 and Heckmann, 2001). The green lines in **(B,C)** indicate reference ranges for unrestricted PRQ (u-PRQ) values obtained from 24-h measurements in adults (according to Heckmann 2001). The values with error bars (standard error of the mean) in **(A,B)** refer to a study by Breithaupt et al. (1980) involving children (*n* = 47) and adults (*n* = 50). **(C)** Findings of Heckmann (2001) that the u-PRQ shows large inter-personal variability and can be classified into subgroups (each color represents a subgroup of 7 in total, subgrouping according to the mean PRQ over 24 h; 1: PRQ > 6.75, *n* = 18; 2: PRQ = 6.25–6.75, *n* = 11; 3: PRQ = 5.75–6.25, *n* = 11, 4: PRQ = 5.25–5.75, *n* = 15; 5: PRQ = 4.74–5.25, *n* = 10; 6: PRQ = 4.25–4.75, *n* = 13, 7: PRQ < 4.25, *n* = 12). **(D–F)** Circadian PRQ variability for different diseases (data extracted from Heckmann, 2001; u-PRQ values, green lines: reference range for healthy adults). **(G–I)** Example of the subgrouping effect for functional PRQ (f-RRQ) values obtained during an experiment with three different exercise strengths. Data extracted from Bräuer et al. (1973); **(G)** 1: men (m), 2: m; **(H)** 1–2: m, 3: women (w); **(I)** 1–4: w. **(J)** Subgrouping effect of PRQ reactivity (f-PRQ values dependent on three conditions: supine, standing, and exercise). Data extracted from Suchantke (1951). **(K)** Relationship between the pulse pressure (PP) and the f-PRQ values during standing for healthy controls (*n* = 56) and subjects with autonomic dysfunction (AD) (*n* = 105) experiencing bradycardia during standing as well ones with tachycardia. The PR-PRQ mapping enables these three groups to be separated efficiently, indicating that the cardiovascular reactivity is different for each group. Data extracted from Weckenmann (1981). **(L)** Changes in the PRQ due to physical exercise of different intensities. The f-PRQ measurements have been obtained from men (*n* = 21, blue dots) and women (*n* = 20, red dots). 1: baseline, 2: FIGURE 3exercise, 3: recovery. Data extracted from Bräuer et al. (1973). **(M)** Changes in heart rate (HR), respiration rate (RR), and PRQ during an orthostatic reactivity test with two groups of subjects (healthy controls, *n* = 57, and subjects with AD, *n* = 105). In case of AD, a characteristic increase of the PRQ while standing can be observed. The HR dynamics showed a subgrouping effect with a strong and a medium increase in HR while standing. Data extracted from Weckenmann (1975). **(N)** Illustration of two functional relationships: the probability of PRQ normalization (PRQ ® 4:1) as a function of the human activity state, and PRQ reactivity (i.e., adaptability of the PRQ to a task or stimulus) as a function of the human health state. **(O)** Correlation between the PRQ (mean of PRQ values from 9:00 to 11:00 am) and the Horne-Östberg index, an index associated with the chronotype of a person. So-called “morning types” showed a PRQ > 4.5, and “evening types” showed < 4.5. It could not be inferred from the publications whether the PRQ used in the study was a rs-PRQ or u-PRQ. Data extracted from Breithaupt et al. (1978).

All three basic types of PRQs (rs-PRQ, u-PRQ, and f-PRQ) depend on the age of the subject. The following studies investigated this topic:

- Breithaupt et al. (1980) published a study about circadian (with a period of about 24 h) changes of the rs-PRQ in five age groups (1: 6–7 years, number of subjects (*n*) = 8; 2: 8–9 years, *n* = 17; 3: 10–11 years, *n* = 10; 4: 12–13 years, *n* = 12; 5: 19–35 years, *n* = 50). It was discovered that the *shape* of the circadian change was age-dependent with a rs-PRQ minimum centered around mid-day for the age groups 1–3, a missing of a circadian change of age group 4 (confirmed by an own statistical analysis using the published data), and a typical circadian rs-PRQ curve for adults (age group 5) with a minimum during night and a maximum around noon (see Figure 3A). Puberty (age group 4) seems therefore to be a turning point with regard to the shape change of the circadian rs-PRQ variability. It is currently not known whether the circadian u-PRQ and f-PRQ changes also show this age-dependent behavior since no study about this has been performed to date. The dependence of the rs-PRQ on age has also been investigated in a study on 326 school children. However, an opposite trend to that reported by Breithaupt et al. was found: while children with a mean age of 7.3 years had a mean rs-PRQ of 4.41, those with a mean age of 13.8 years had a mean rs-PRQ of 4.23 (von Zabern, 2001). However, the study did not differentiate between the sexes and did not test whether the age-dependent PRQ differences were statistically significant.
- In a remarkable study published in German in the 1990s, Matthiolius and Hildebrandt (1995) reported the results of 6,677 single PRQ measurements on 1,820 school children (age range: 5.5–15 years). The study investigated how the daily rs-PRQ and f-PRQ (PRQ during standing) depend on age and sex. It was found that there is (i) an increase of the rs-PRQ, f-PRQ, PRQ difference between standing and supine postures (f-PRQ-rs-PRQ) and a variability of the PRQ during each measurement session with age and (ii) a specific relationship between both PRQ parameters with sex (for a detailed discussion of this aspect, see section “The PRQ Is Sex-Dependent”). These findings demonstrate well the changes of the cardiorespiratory state during human development.
- How the D-rs-PRQ behaves with respect to the whole human lifespan was investigated by Stöhr in the 1980s based on HR, RR, and PRQ values published in different studies. He concluded that there is an increase in the PRQ during childhood until about 20 years and a subsequent decline (Stöhr, 1985) (for a discussion of this finding, see also Hildebrandt, 1994).

### The PRQ is Time-Dependent: Ultradian, Circadian, and Infradian Rhythms

The PRQ of a human depends on the time when it is measured due to the chronobiological variability of human physiology. The *circadian* oscillation of the PRQ has been reported and investigated in several studies (Hildebrandt, 1954, 1955, 1976; Engel et al., 1969; Breithaupt et al., 1980; Heckmann, 2001). The circadian oscillation found and investigated was nycthemeral, i.e., related to the day-night cycle. The main findings about the circadian oscillation can be summarized as follows:

- As discussed already in the previous section (The PRQ Changes During Human Development), the circadian oscillation of the PRQ is age-dependent with respect to the amplitude and phase (Breithaupt et al., 1980).
- The *amplitude* of the circadian oscillation is smaller for the rs-PRQ than for the u-PRQ (Breithaupt et al., 1980; Heckmann, 2001).
- There is a large *intersubject variability* of the amplitude of the circadian oscillation of the u-PRQ (Heckmann, 2001) and rs-PRQ (Engel et al., 1969).
- Pathophysiological states in humans are reflected by changes in the typical circadian PRQ variability (Heckmann, 2001).
- It has been shown that a large amplitude of the circadian PRQ oscillation can be reduced in subjects by administering a barbiturate or, preferably, by health interventions, e.g., balneology treatments (Hildebrandt, 1954, 1955, 1980).
- The *phase* of the circadian PRQ oscillation is associated with the subject-specific chronobiological default state (the chronotype; assessed by a respective questionnaire). The morning PRQ can be used as an index to determine the specific chronotype (Breithaupt et al., 1978) (see Figure 3O).

Regarding *infradian* (longer than a day) oscillations of the PRQ, we were unable to find any systematic investigation. However, a PRQ oscillation with a period of approximately a week can be inferred from a figure published by Hildebrandt (Hildebrandt, 1980) based on the data of Fechner (1980), and in one study, long-term changes of the PRQ in humans were documented along with correlated changes in atmospheric air pressure (Hildebrandt, 1955).

**Figure 4:**
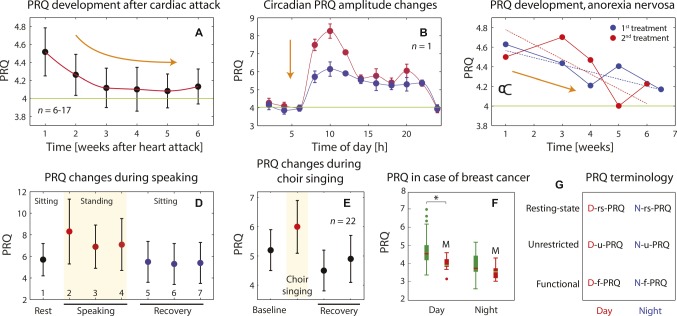
**(A)** Dependence of nightly resting-state PRQ (N-rs-PRQ) values as a function of time after a cardiac attack (*n* = 6–17, dependent on data point, mean ± standard error of the mean; data extracted from Kümmell and Heckmann, 1987). The PRQ normalization toward 4:1 is evident during the recovery phase after the incident. **(B)** The effect of a health intervention [12 spa (balneology) treatments] on the circadian PRQ oscillation amplitude is shown exemplarily for one subject. A less pronounced morning and evening PRQ peak is visible, indicating a normalization of the physiological state. Mean and standard error of the mean values are shown based on averages over 3 days. It was not possible to infer from the publication whether the PRQ values represent rs-PRQ or u-PRQ. Data extracted from Hildebrandt (1955). **(C)** N-rs-PRQ values for one subject (female) with anorexia nervosa that obtained two treatment periods (red and blue curves) over several weeks. Data extracted from Heckmann et al. (1990). The PRQ values represent monthly daily resting-state PRQ (D-rs-PRQ) values. A clear trend of PRQ normalization toward 4:1 is visible during the course of the treatments. **(D)** Changes of functional PRQ (f-PRQ) values during an experiment with three types of speaking tasks: 1: baseline; 2: speaking, style 1: recitation; 3: speaking, style 2: declamation; 4: speaking, type 3: conversation; 5-7: recovery after 2; recovery after 3; recovery after 4. Tasks 1 and 4–6 were performed while sitting, tasks 2–4 while standing. The changes of f-PRQ are therefore due to speaking and changes in the body position in parallel. Data extracted from Von Bonin et al. (2001). **(E)** Effects of choir singing on the f-PRQ (Marti, 2012). **(F)** PRQ differences between two groups of breast cancer patients: with (M, *n* = 11) and without (*n* = 26) metastasis. The PRQ values there represent mean unrestricted PRQ (u-PRQ) ones calculated for the night (1:00–5:00 am, N-u-PRQ) and the day (5:00–1:00 am, D-u-PRQ). Data extracted from Bettermann et al. (2001). **(G)** Schematic representation of the PRQ terminology proposed and employed in this manuscript.

*Ultradian* PRQ oscillations (shorter than a day) are also a feature of PRQ variability. During sleep, ultradian PRQ changes can be observed (for a discussion, see section “The PRQ Shows Specific Patterns During Sleep”). During the day, ultradian oscillatory features of the circadian PRQ variability can be seen in the figures of a few studies (e.g., Figures 3A–F, 4B), but no specific investigation of them has been performed to data to the best of our knowledge.

### The PRQ Shows Specific Patterns During Sleep

The PRQ change during sleep has been investigated by several studies, yielding these main findings:

- While children (6–12 years) show a N-rs-PRQ during nightly sleep in the range of about 4.5–5, adults tend to have N-rs-PRQ values around 4 (the so-called *nightly PRQ normalization*) (Pöllmann and Hildebrandt, 1970; Breithaupt et al., 1980; Heckmann, 2001). Newborns during quiet sleep often exhibit long phases of PRQ values around 3 (Hoyer et al., 2001). The N-rs-PRQ is therefore age-dependent (see Figure 3A). Data for older people (>70 years) are, to the best of our knowledge, not yet available.
- The time when the PRQ normalization during sleep is most likely to occur seems to be a matter of how the PRQ is determined: while around 3:00 am is found when analyzing the data with respect to the clock time (Hildebrandt, 1976), the last phase of sleep (last sleep hours) is found to show the highest probability of the PRQ normalization (Pöllmann and Hildebrandt, 1970) (see Figure 2C), indicating that the individual “biological time” is important regarding this aspect. When interpreting N-PRQ values, it seems therefore important to distinguish between the two time reference frames, i.e., the *clock time* and the *biological time* (i.e., the time relative to a physiological event, e.g., the time of falling asleep). This aspect probably is related to the sleep architecture, which follows a specific pattern (cyclic change between non-REM and REM sleep).
- The total minimum of N-rs-PRQ variability was found to occur about 30 min before awakening (Pöllmann and Hildebrandt, 1970) (see Figure 2C).
- The type of daily activity determines the dynamics of the N-rs-PRQ during the night (Hildebrandt, 1955): high cognitive activity during the day in combination with low physical exercise causes a low PRQ level during the night, while a high level of physical exercise is associated with a higher overall PRQ level and more fluctuations during night. The N-rs-PRQ level is positively correlated with the 24-h circadian u-PRQ amplitude (Zerm et al., 2008), i.e., the higher the circadian u-PRQ oscillation amplitude, the higher the N-rs-PRQ value.
- The probability of the PRQ normalization occurring during the last hours before awakening (N-rs-PRQ → 4) was found to be independent of the amount of physical exercise (8 × 45 min sessions on a bicycle ergometer throughout the day with different amounts levels of difficulty) during the preceding day (Pöllmann and Hildebrandt, 1970).
- The N-rs-PRQ depends on the sleep stages in adults: while light and deep sleep phases showed PRQ values around 4, a value of around 3 was found in the REM phase (Bartsch et al., 2007). This study only analyzed the PRQ phases that showed a constant PRQ over a specific period (frequency coupling) and also a phase entrainment between HR and RR (phase coupling) during this time. During sleep, a complex correlation between the HR and the RR is evident with the PRQ capturing one specific aspect. In a study investigating the N-rs-PRQ with a detailed division of the sleep stages (five stages), Zerm et al. (2008) showed that there is a positive correlation between the N-rs-PRQ and the sleep depth, i.e., the higher the depth of sleep, the lower the N-rs-PRQ. In this study, sleep depth was defined according to the scale “awake → REM → non-REM I–II → non-REM III–IV.” A lower N-rs-PRQ value during deep sleep than REM sleep (3.83 ± 0.59 vs. 4.12 ± 0.74) was also found by von Bonin et al. (Von Bonin et al., 2014). These two findings seem to contradict the finding of Bartsch et al.; however, Bartsch et al. determined the N-rs-PRQ only when there was a phase entrainment between HR and RR – in contrast to Zerm et al. who determined the N-rs-PRQ continuously.
- The subjectively perceived sleep quality correlates negatively with the mean N-rs-PRQ, i.e., subjects rated the quality of their sleep lower when the N-rs-PRQ was higher (Zerm et al., 2008).
- Pathophysiological states in humans are reflected in characteristic deviations of the N-rs-PRQ values with respect to the normal ranges for healthy individuals (Hildebrandt, 1988; Heckmann, 2001). How the PRQ changes during night is therefore related to the overall physiological state of the individual.

### The PRQ Changes With Behavior and Body Posture

That the PRQ changes with behavior (e.g., physical activity) and body posture was noticed and studied already in the first phase of the PRQ research history. Several studies investigated how these two factors determine the PRQ. Concerning the impact of the physical activity on the PRQ, the following main findings were obtained:

- An increase in physical activity causes an *increase* or a *decrease* in the f-PRQ. The direction seems to be mainly linked to four factors: the *amount of physical activity*, the *time pattern* between exercise and rest, the individual *physiological constitution* (see section “The Individual Physiological Constitution Is Linked to the PRQ”), and the *sex* (see section “The PRQ Is Sex-Dependent”).
- In a first systematic study conducted already in the 1960s, Hildebrandt and Daumann found that while physical exercise of different degrees always causes an increase in HR and RR, the f-PRQ generally exhibits a drop and the f-PRQ distributions change depending on the physical load (Hildebrandt and Daumann, 1965). With a study population of 21 men, the authors showed that while the f-PRQ values of subjects in the supine body position and sitting were in the ranges of 2.5–5.5 and 3.5–6, respectively, the physical exercise (30 min) caused a narrowing of the distributions and more centering to integer values. Interestingly, the more strenuous the exercises, the more the f-PRQ centered around a value of 4, indicating the occurrence of a PRQ normalization due to the strenuous physical exercise. In a study testing the effect of a short period (2 min) of exercise, PRQ was found to decrease (Weckenmann et al., 1988).
- Bräuer et al. (1973) extended the study design by having subjects perform physical exercise (with three levels of difficulty). Then they analyzed various factors, especially the source of the intersubject variability. The study found that the PRQ baseline values as well as the magnitude and sign of the exercise-induced f-PRQ changes were dependent on the sex. Men had a higher baseline PRQ level (than women) and showed generally a drop in f-PRQ during the tasks, while women showed a slight increase (see Figure 3L). Furthermore, it was found that the intersubject variability is explained by dividing the subjects into three groups based on their similar f-PRQ dynamics over the whole experiment. Group 1 showed a completely different pattern than groups 2 and 3 with respect to the f-PRQ changes due to the most exhausting exercise. The exercise initiated a *decrease* in f-PRQ for group 1, with a tendency to reach f-PRQ values toward 4 (similar to the finding of Hildebrandt and Daumann, 1965), while subjects of groups 2 and 3 showed a PRQ *increase* during this type of exercise (see Figures 3G–I). An analysis of the reproducibility of the results obtained revealed that the f-PRQ values during the less demanding exercise were most reliably replicable – even more so than the rs-PRQ values. The study of Bräuer et al. is important since it highlights the need to perform a subgroup analysis of the results based on the f-PRQ pattern observed and to analyze the data separately for men and women.
- The extent of physical activity over the day determines the amplitude of the circadian PRQ oscillation in a *time-dependent non-linear* way: physical exercise every 4 h reduced the circadian amplitude, while exercises every 2 h increased it (Engel et al., 1969). The time pattern between exercise and rest, and possibly, the exercise dose (i.e., the magnitude × time) therefore also seems to play a role modulating the PRQ response due to physical activity.
- Physical activity is linked with cognitive activity. Changes in physical and cognitive activity in parallel are also reflected by f-PRQ changes. This has been shown, e.g., to be the case during speaking (causing an increase in f-PRQ depending on the type of speech) (Von Bonin et al., 2001), cognitive activity (concentrating alertness on an abstraction vs. listening to a spoken text) (Marti, 2013), and choir singing (Marti, 2012) (see Figures 4D,E).

Another factor determining PRQ values is the body posture. It is not only related to behavior and physical activity but also independent of it. While physical activity normally involves changes in the body posture, a specific body posture does not automatically involve physical activity. The following insights about the relationship between the PRQ and the body posture have been gathered so far:

- The relationship between PRQ and body posture is time-dependent, i.e., it is important to distinguish three regimes. These are PRQ changes during (i) the *transition phase* from changing one body posture to another, (ii) the *adaptation phase* (the phase when the body physiology adapts to the new state by actively regulating diverse parameters), and (iii) the *stabilization phase* (the phase when the regulation mechanism establishes a new stable state). All phases are linked to a functioning adaptability (reactivity) of the CRS and CVS to body posture change or different prolonged body posture states. The PRQ studies investigating the body posture effect so far analyzed only the stabilization phase – most probably due mainly to technical reasons since the analysis of the PRQ changes during phases 1 and 2 needs a sufficient time resolution, which was not available. Although this limits the insights into the relationship between the PRQ and the body posture, the results obtained related to the stabilization phase are already significant.
- The change from the supine position to standing is generally associated with an increase in the PRQ, with the magnitude of increase depending on the individual subject. The magnitude of PRQ increase is mainly determined by how well the orthostatic cardiovascular regulation works (orthostatic tolerance). In normal *healthy* humans, the transition from supine to standing is associated with an increase in pulse pressure (difference between systolic (SBP) and diastolic (DBP) blood pressure: PP = SBP–DBP), HR and PRQ (Suchantke, 1951; Weckenmann, 1975, 1981) (see Figures 3J,M).
- Already in 1950, Suchantke (1951) discovered by measuring f-PRQ values based on a functional task (supine → standing → physical exercise) that the intersubject variability could be explained by the presence of different PRQ reactivity patterns linked to four groups. According to his findings, each group is characterized by a specific rs-PRQ value and a specific magnitude of f-PRQ change during the tasks. Subjects with a high rs-PRQ in the supine position (about 6) tended to also have a large PRQ change when shifting to a standing position, indicating an orthostatic intolerance (see Figure 3J).
- Weckenmann investigated this aspect further and revealed that subjects with an orthostatic intolerance, i.e., an *autonomic dysfunction* (AD), (i) show a different relationship between the PP and the f-PRQ during standing compared to healthy controls (Weckenmann, 1981) (see Figure 3K) and (ii) have a higher f-PRQ increase when changing from supine to standing compared to healthy controls (Weckenmann, 1975) (see Figure 3M). While subjects with AD could be divided into two groups according to their HR during standing (group 1: bradycardia; group 2: tachycardia), the PRQ always showed a standing-induced increase independent of the two groups (Weckenmann, 1975). In parallel, the two groups could be separated by comparing the standing f-PRQ with the standing PP (Weckenmann, 1981) (see Figure 3K). The relationship between the f-PRQ values during the supine position with those during standing is also different in subjects with AD compared to healthy ones (Weckenmann, 1982).

### The PRQ Is Linked With Psychophysical and Cognitive Activity

There are only a few published studies reporting investigations into how psychophysical parameters or cognitive activity is linked to the PRQ. Marti (2013) observed PRQ changes during cognitive tasks (concentrating alertness on an abstraction vs. listening to a spoken text), and Hildebrandt and Engel (1963) found a statistically significant positive correlation (*r* = 0.45) between fluctuations in auditory reaction times of humans with fluctuations of the PRQ. Interestingly, the HR and RR fluctuations correlated to a lesser extent than the PRQ fluctuations.

### The PRQ Is Sex-Dependent

A further aspect of the PRQ is that it depends on sex. This fact, however, was unfortunately not considered in several PRQ studies published. There are two main studies that showed the sex-dependence of the PRQ:

- In a large study by Matthiolius and Hildebrandt (1995) investigating the age-dependence of the D-rs-PRQ and D-f-PRQ in girls and boys (*n* = 1,820; age range: 5.5–15 years), a clear *sex-dependent trajectory* of the *age-dependent* D-rs-PRQ and D-f-changes was obtained, with higher PRQ values for boys in the age range of approx. 5.5–9 years and for inverse relationship in the age range of approx. 13–15 years. This effect was evident for both body postures (standing and supine). Also, the PRQ differences between standing and supine positions, as well as the variability of PRQ values per age class, showed different sex-related patterns during development (Figure 2B).
- A sex-related effect on D-rs-PRQ and D-f-PRQ values for adults (21 men, age: 30–34 years; 20 women, 20–50 years) was observed in a study investigating the effect of physical exercise on PRQ changes (Bräuer et al., 1973). Compared to males, females (i) had statistically significantly lower rs-PRQ values at baseline and (ii) showed a statistically significantly stronger PRQ increase during the exercise (Figure 3L). In addition, the reproducibility of PRQ measurement was found to be better for women than for men.

These findings are not unexpected since there are differences in physiology between both the sexes (Legato et al., 2016; Boese et al., 2017; Kerkhof and Miller, 2018). A sex-dependence was found by many other studies investigating the CRS and CVS in humans. For example, parameters of heart rate variability (HRV) (Sinnreich et al., 1998; Antelmi et al., 2004; Park et al., 2009; Boettger et al., 2010; Nemati et al., 2013; Hernandez et al., 2015), blood pressure (Kuo et al., 1999; Harrap et al., 2000; Ellis et al., 2004), and respiration (Snieder et al., 1997; Becklake and Kauffmann, 1999; Carey et al., 2007) depend on sex. Cardiac activity is more dominantly regulated by the parasympathetic nervous system in women, while for middle-aged men, the regulation is more due to the sympathetic nervous system (Kuo et al., 1999). Also, the exercise vasodilator response is different between the sexes with a greater one in females (Kellawan et al., 2015). The aging of the CVS is also sex-dependent (Kane and Howlett, 2018). These factors are clearly associated with the individual PRQ.

### The Individual Physiological Constitution Is Linked to the PRQ

Despite the fact that (age- and sex-adjusted) normal or reference ranges for physiological variables are widely used in human physiology and medicine, it is known that the variable values from a large population follow multimodal distributions (i.e., distributions with several peaks), indicating a subgrouping of the subjects (Zaitseva and Son’kin, 2005). One factor explaining this is the existence of subgroups according to the individual *physiological constitution,* i.e., the specific inborn integrated physiological (and anatomical) phenotypic traits determined by genetic inheritance and epigenetics.^2^ While the existence of constitution types is well supported by empirical evidence, the classification (number and defining characteristics) is still a matter of research and debate, with several different models posited to classify the proposed constitutional types (Günther, 1922; Ciocco, 1936; Conrad, 1941; Portmann, 1962, 1969; Kim et al., 2009; Wang, 2012; Pham et al., 2013; Metzger, 2016; Gruber, 2017; Yu et al., 2017). That the physiological constitution partially may contribute to the *disposition* for disease was a finding of numerous studies (e.g., Feigenbaum and Howat, 1934; Koleva et al., 2002). A specific aspect of the physiological constitution is the prevailing state of chronobiological rhythms of a subject, termed “chronotype” (Bender and Wellbery, 1991).

The current, actual, physiological constitution of a human being can be regarded as a combination (and interaction) of the specific inborn constitution type combined with the anatomical and physiological states and changes experienced during that individual’s life until now. A term encompassing both these aspects of intersubject variability is simply the “phenotype.” The phenotype-based classification of human physiological states is an intensive research field in the context of personalized medicine. A research field is generally termed “phenomics” (Houle et al., 2010).

That the physiological constitution also plays a role in the PRQ of humans has been shown by several studies:

- As already mentioned in section “The PRQ Changes With Behavior and Body Posture,” Suchantke (1951) observed as early as the 1950s that the f-PRQ values during a functional task (supine lying → standing → physical exercise) showed different trajectories in the population investigated (based on about 2,300 single measurements) that could be classified into four groups (see also section “The PRQ Changes With Behavior and Body Posture”). He linked these groups to four physical constitutional types (group 1: healthy normal; group 2: subjects with rigid CRS reactivity and overactive nervous system; group 3: subjects with a tendency to have metabolic diseases; and group 4: subjects with an overactive CR reactivity and a dissociation of the physiological subsystems, i.e., an uncoordinated interplay of the diverse physiological processes) according to the f-PRQ values depending on the functional task. The associated PRQ values were (supine → standing → exercise): group 1: 4.0 → 4.1 → 4.2; group 2: 2.5 → 2.9 → 3.5; group 3: 5.6 → 5.7 → 7.0; and group 4: 5.5 → 7.0 → 8.0. These four types were linked to constitutional and pathophysiological phenotypes.
- Also in a study employing a functional PRQ test (physical exercises of different strengths with rest periods between), Bräuer concluded that he could subgroup the PRQ reactivity profiles for the subjects according to the characteristic PRQ patterns (Bräuer et al., 1973). The pattern similarity was assigned to three groups, and the authors concluded that these groups seem to have different physical constitutions (Figures 3G–I).
- Von Bonin et al. (2001) introduced 2001 the *constitution PRQ* (“Konstitutions-QP/A” in German; in the following termed *c-PRQ*), i.e., the mean PRQ value of the first non-REM sleep phase (in general, the first one during sleep) of a subject. The c-PRQ is a specific type of N-rs-PRQ that, according to the authors, captures in particular the maximally possible trophotropism of the individual, i.e., state of maximal body function harmonization in the sense of an optimal integration of physiological rhythms and processes, metabolic anabolism and regeneration during rest, opposite to ergotropism, the state of maximal alertness, katabolic metabolism, and readiness to react to external stimuli. The study found that the relationship between the c-PRQ values and the D-rs-PRQ during sitting was subject-specific and divided the population into two groups: one with D-rs-PRQ ≈ N-rs-PRQ (subjects with a well-functioning physiological state) and the other with rs-PRQ > c-PRQ (subjects with a phenotype indicating a sub-optimal physiological state). The c-PRQ values averaged over both groups were 4.47 ± 0.97 (own calculation based on the data given in the paper). In a follow-up study, Von Bonin et al. (2014) showed that the ratio of the D-rs-PRQ to the c-PRQ (D-rs-PRQ/c-PRQ), termed “day-night-index-heart respiration ratio” (DNI-HRR) by the authors, was associated with the subjectively perceived “general health” and “mental well-being” of the subjects (*n* = 87). The study states “participants with a DNI-HRR < 1.06 felt healthier, indicating better mental well-being and less depressive moods.” This study indicates that a good way to derive a PRQ parameter that captures the individual constitution is to determine the D-rs-PRQ and to divide it by the mean PRQ value of the first non-REM sleep phase.
- As already mentioned in section “The PRQ Is Time-Dependent: Ultradian, Circadian, and Infradian Rhythms,” the chronobiological trait of a subject is also linked to the PRQ. The *phase* of the circadian PRQ oscillation is associated with the chronotype, as shown by Breithaupt et al. (1978) (Figure 3O).
- A link to *anatomical* aspects of the physiological constitution has been shown by the work of von Zabern. In two studies, he investigated a possible relationship between the D-rs-PRQ and the head form of infants (von Zabern, 1970, 2001). In particular, he showed that the age- and sex-corrected head circumferences of children correlated with the PRQ. Children with a larger than normal head circumference had also larger PRQ values (4.40) compared to those with smaller than normal ones: PRQ: 4.84 vs. 4.44 (von Zabern, 1970) and 4.40 vs. 4.12 (von Zabern, 2001), respectively. Children with larger heads had a higher HR and lower RR than those with smaller ones (von Zabern, 1970, 2001).

## Medical Aspects of the PRQ: Application for Disease Classification and Monitoring

### The PRQ as an Indicator for Health and Disease

It has been obvious since PRQ research was first conducted in the 1920s that the PRQ is relevant in assessing the status of human health and disease. Studies showed that a person’s pathophysiological state is indicated by deviations of the PRQ from normal values during static (e.g., resting) or dynamic states (e.g., body posture transitions). Besides this, interpreting PRQ based on its chronobiological variability has been shown to offer new insights into the health and disease states of humans. In particular, the following insights have been gained with regard to the PRQ as an indicator for health and disease:

- The age-adjusted D-rs-PRQ (mean over 1:00–5:00 pm) has been shown to be statistically significantly *lower* for breast cancer patients with metastasis (*n* = 11) than those without (*n* = 26) (Figure 4F; Bettermann et al., 2001). This difference was not present for the age-adjusted N-rs-PRQ, nor was there a difference found in PRQ values between healthy subjects and those with either breast cancer or diabetes. With an extended analysis of the CRI characteristics, the authors also showed that in patients with metastasized breast cancer “relevant phase locking pattern classes with ratios greater than 4:1 were significantly less predominant and/or cyclically stable” (Bettermann et al., 2001). These findings are in line, e.g., with findings that the survival rate of cancer patients is linked to the degree of deviations of the HRV, and thus the CRS/CVS, from normal reference ranges (Kloter et al., 2018). In a case report about a breast cancer patient (age: 37 years), a *low* D-rs-PRQ of 2.5 was also noted (Göbels and Allmer, 2014), which backs up the finding of Bettermann et al. *Higher* D-rs-PRQ and N-rs-PRQ values (compared to reference intervals of healthy subjects) have been found in patients with an acute myocardial infarction and hyperthyroidism (Heckmann, 2001).
- That the circadian variability of the rs-PRQ is altered by disease has been demonstrated for the case of myocardial infarction (almost no variability of the PRQ during the whole circadian cycle) and for hyperthyroidism (pronounced ultradian fluctuations) (Figures 3D–F; Heckmann, 2001).
- As already explained in section “The PRQ Changes With Behavior and Body Posture,” a functional test involving posture transition and/or changes between rest and physical exercise is a sensitive tool to test for the presence of an orthostatic intolerance, and thus AD, in a subject. Too high PRQ reactivity due to the task (*functional over-reactivity*) and too low reactivity (*functional rigidity*) are both associated with pathophysiological states (Weckenmann, 1975, 1981).

### The PRQ as a Useful Tool for Disease and Treatment Monitoring

Testing how the PRQ behaves as a marker for disease and treatment monitoring was an aim of several studies so far, with the following main findings:

- The changes of the circadian u-PRQ *amplitude* can be employed as a marker for treatment monitoring. This has been shown by the group of Hildebrand, demonstrating, e.g., that repeated health treatments with balneology (therapeutic bathing) over several weeks caused a decrease in the amplitude, indicating a normalization process of the CVS/CRS and the success of the health intervention (Figure 4B; Hildebrandt, 1955). The effect has been interpreted as an “adaptive normalization” of physiology due to a regulation and stimulation therapy (Hildebrandt and Gutenbrunner, 1996).
- In addition, the changes of the *acrophase* of the circadian u-PRQ oscillation are relevant for disease monitoring. While at the beginning of disease, the acrophases of HR, RR, and PRQ are often not aligned and show values deviating from the reference ranges, during the healing phase, realignment occurs, with a final acrophase concentration at around 3:00 pm (Rippert, 1984; Hildebrandt, 1985).
- After a cardiac arrest, a characteristic change of the N-rs-PRQ (restoration of the N-rs-PRQ normalization toward a value of 4) normally occurs in patients, reflecting a healing process (Figure 4A; Kümmell and Heckmann, 1987).
- The treatment progress of anorexia nervosa was shown to be associated with N-rs-PRQ normalization over several weeks (Figure 4C; Heckmann et al., 1990). This pattern was shown for two treatment periods in a single female.
- A D-rs-PRQ normalization taking place during the treatment (massage therapy) of a breast cancer patient (age: 39 years, mastectomy, no metastasis, no chemotherapy but mistletoe therapy received) has been documented in a case report (Göbels and Allmer, 2014). During the course of the treatment (14 massage therapy sessions), the initial low D-rs-PRQ (2.5) increased, reaching a value of 3.6 after the least treatment. This indicates that a PRQ normalization process took place. Interestingly, the body temperature difference (rectal – oral) showed a similar trend during the treatments (large difference at the beginning and normalization at the end).
- It has been reported that the prognosis for cancer patients seems to be associated with the specific pattern of f-PRQ values obtained by a functional test (supine → standing → exercise). Cancer patients with a good prognosis tended to show values around 5.6 → 5.7 → 7.0, while those with a poor prognosis showed a pattern of around 2.5 → 2.9 → 3.5 (Suchantke, 1951). This is similar to the finding of Bettermann et al. (2001) that saw low D-rs-PRQ values and a reduced dynamic range of the PRQ in breast cancer patients with metastasis (compared to those without) (Figure 4F).
- Measuring the daily D-rs-PRQ in 14 patients with influenza during the course of the disease, Müller (1972) found a tendency of PRQ normalization toward 4:1 during the disease development. Furthermore, patients that had a higher PRQ than 4 at the beginning of the disease were found to heal faster than those with a PRQ of less than 4. The latter had a prolonged healing phase and a tendency toward chronic disease progression. Hildebrand commented that this finding was in line with his own observations that patients with a higher PRQ at disease onset were more likely to have a fast regulating ergotropic phenotype, while those with a low PRQ were associated more with a slower regulating trophotropic phenotype (Hildebrandt, 1986).

## Case for Reviving the Use of the PRQ for Basic Research About Human Physiology and for Applications in Medicine

Based on the literature reviewed, we believe the PRQ has a great potential for use in future studies about human physiology as well as for applications in medicine. The case for its revival as an informative biological marker can be summarized by these three reasons:

1. As an intrinsic property, the PRQ captures a fundamental aspect of the CRI, the relationship between two constantly active oscillatory systems (cardiac and respiratory system) with respect to their frequencies. This frequency relationship, i.e., the PRQ, represents the current adaptive state of the CVS and CRS to internal and external states and demands. As such, the PRQ includes relevant information for a qualitative and quantitative assessment of the physiological state of an organism, especially its cardio-respiratory adaptability due to changes in state. The PRQ is therefore suited to studies and applications requiring a parameter to capture a fundamental aspect of CRI in an organism.
2. The measurement of the PRQ does not require sophisticated equipment, and the calculation is simple, involving no need for adjustable parameter values. As discussed in section “Measurement of the PRQ,” the PRQ can be directly calculated, e.g., based on the ECG signal alone by extracting the respiration-related information (i.e., the ECG-derived respiration – EDR).
3. Since the PRQ is not a “new” parameter, but a “re-discovered” one, a lot of knowledge about the PRQ has been accumulated over the last decades. This knowledge, as reviewed here, can be used as a reference for comparison to new PRQ measurements and findings, i.e., research about the PRQ does not have to start from scratch but can build on the already impressive insights regarding the PRQ.

## Recommendations for the Use of the PRQ in Studies and Applications

Based on the literature reviewed, we make the following recommendations to ensure proper use of the PRQ in studies and for medical applications:

- *Use the appropriate PRQ terminology.* There are different subtypes of the PRQ, which are specified in the PRQ-related terminology introduced in this article (section “The PRQ: Definition and Terminology,” see also Figure 4G). We recommend using this terminology when reporting newly conducted studies, as well as when discussing previously conducted studies. This will help to minimize the risk of incorrectly comparing different PRQ findings due to misinterpretation of the PRQ findings. This is particularly important for researchers when interpreting PRQ study results compared to those reported in existing literature since the term “PRQ” has often been used in a context-dependent way and without differentiating between different subtypes of PRQ parameters (section “The PRQ: Definition and Terminology”).
- *Use an appropriate study protocol when employing the PRQ*. The PRQ is intrinsically a sensitive parameter to the state and state changes of an organism, making it also easily influenced by factors that should not influence the study. For example, when performing rs-PRQ studies, the subject must have enough time to reach a stable physiological state (by, e.g., letting subjects lie on their back for 10 min covered with a blanket for comfort). It is also important to avoid any inconvenience or disruption for the subject during the experiment. The requirements for bringing a subject into a relaxed, not stressed state are dependent on the individual subject, i.e., it must be ensured that subject-specific requirements are met. Furthermore, the study design needs to be optimized in order to capture either task-unrelated physiological states (e.g., the rs-PRQ at different time points during the day) or functional changes with a PRQ reactivity test (e.g., a change of body posture). Study design should also consider that the sequence of tasks in a protocol can have an influence on the results. This is because the current state of body physiology depends partially also on past states, causing “carry-over effects” that can make data analysis difficult and can mask the effects the study was looking for. When designing (and analyzing) functional PRQ experiments, the concepts already developed for neuroimaging studies could be also applied. In particular, the concept of the “mixed block/event-related design” (Visscher et al., 2003; Petersen and Dubis, 2012) could be employed to differentiate between transient changes in the PRQ from PRQ states that are linked to a sustained CVS/CRS activity state.
- *Take into account that the PRQ is influenced by age*, *sex*, *chronobiology*, *body posture*, *behavior*, *physiological constitution*, *environmental influences,* and the *health/disease state.* That the PRQ depends on these main factors is important to consider when creating the study protocol as well as when analyzing and interpreting the PRQ data. Failure to do so can lead to false conclusions. Especially, the variables such as age, sex, and chronobiology need to be considered. While age is generally considered a variable in human physiology studies, “sex differences between women and men are often overlooked and underappreciated” (Boese et al., 2017). The same is also true for chronobiology (e.g., the circadian phase), which is also often not well considered in human physiology studies. In addition, outdoor temperature and previous meals have an effect on the physiology that influences studies about the cardiorespiratory system (Goldstein and Shapiro, 1996; Madaniyazi et al., 2016). Studies about the PRQ need to take these mentioned factors into account.
- *Take into account that the PRQ is linked to the chronobiology state in a complex way*. The time dependency of the PRQ is a very important aspect that needs to be considered whenever the PRQ is measured and PRQ results need to be interpreted. It is an intrinsic property of the PRQ that it depends on the chronobiology of the subject (as reviewed in section “The PRQ Is Time-Dependent: Ultradian, Circadian, and Infradian Rhythms”). When designing a study, it must be defined if the study endpoint is dependent on the chronobiology or not. If it should be independent, it is recommend to measure the subject at the same time of day (e.g., morning) and to consider the remaining chronobiological variability in the statistical model applied (e.g., by using time of day, the experiment was performed as an additional regressor). Ultradian, circadian, and infradian rhythms should always be considered when designing the PRQ studies and analyzing the data.
- *Perform an empirically based subgrouping analysis.* It has been observed by several PRQ studies that the PRQ intersubject variability is not randomly distributed but can be explained by distinct data patterns associated with a subgrouping effect. Such an effect can be detected by clustering methods that separate the data into distinct clusters empirically (Jain, 2010). The clusters could then be analyzed to associate with the main factors such as age, sex, and physiological constitution or to establish whether they represent unique types of individual physiological states and traits.
- *Take into account that the PRQ is lognormally distributed.* The fact that the PRQ follows a lognormal distribution is an important consideration when performing data analysis. Since a skewed distribution can cause errors in parametric statistical tests (Choi, 2016), it is necessary to take the PRQ’s lognormal distribution into account. One way is to log-transform the PRQ values prior to analysis. This ensures the validity of the statistical analysis. However, log-transforming data also have limitations (Zhou et al., 1997; Feng et al., 2014). A better way is likelihood-based or bootstrap-based approaches (Zhou et al., 1997; Feng et al., 2014; Choi, 2016). Unfortunately, in most of the published PRQ studies, the lognormal distribution of the PRQ has not been considered in the statistical testing – aspect readers of these studies need to be aware of when interpreting the statistical analysis results of these studies.
- *Use the PRQ in combination with the other parameters related to the CRS and/or CVS state.* Combining the PRQ measurement with the measurement of the other parameters of the CRS and CVS is a fruitful approach to increase the capture of complex behavior of both systems. For example, the PRQ can be combined with the measurement of blood pressure (as has been done in a few studies already), HRV, body temperature, parameters capturing the electrodermal activity or blood gas concentrations.

## Summary, Conclusions, and Outlook

As we summarized in the previous sections, the PRQ is a powerful, easy-to-measure, easy-to-calculate, and useful parameter that captures basic properties of the complex interaction between the cardiac and respiratory systems, reflecting fundamental properties of human physiology (e.g., chronobiological state, reactivity and adaptability of the CVS and CRS).

The PRQ contains information that cannot be obtained when analyzing the HR or the RR separately. The reason for this is that the PRQ reflects *emergent* properties of the complex interplay between the cardiac and respiratory activities. Even the HRV, which contains also information about the respiration (Brown et al., 1993; Aysin and Aysin, 2006; Lenis et al., 2017), is linked to the PRQ in such a way that the HRV cannot be used as a surrogate for the PRQ. For example, the correlation strength between the PRQ and the HR as well as the ln LF/HF (low frequency/high frequency power ratio) HRV parameter depends on the daytime (higher correlations during day), while the correlation strength between the PRQ and ln LF/HF is generally weak (day: *r* = 0.31 ± 0.09; night: *r* = 0.21 ± 0.06, *r*: Pearson’s correlation coefficient) (Cysarz et al., 2008). One novel information the PRQ contains, which is not available from the HR, RR, and HRV, is, e.g., the already discussed (section “Historical Development, Measurement Methods, Basic Characteristics, and Link to Cardiorespiratory Interactions”) quantization of the PRQ values with preferred values of the harmonic ratios *n*:*m* (with *n* = 3–6 and *m* = 1) and the tendency of the preferred 4:1 coupling (PRQ = 4). The absolute PRQ values thus have already a meaning due to the emergent dynamical order of cardiorespiratory interactions. In other words, the cardiorespiratory system behaves as a self-organized system with the emergence of order, reflected in the PRQ properties.

In our review, there are two factors explaining why the PRQ is *not* widely applied in current studies about human physiology and why it is normally *not* used for medical applications at present. First, the concept of the PRQ is not well known in the scientific community (since modern studies only use it rarely). Second, since much of the knowledge accumulated about the PRQ over the last decades was published in German, it is not readily accessible to the English-speaking scientific community. With the present review article, we address both aspects by giving a concise introduction to concept of the PRQ and summaries of the German publications and findings.

Due to its ability to capture the individual cardio-respiratory state of a person in a unique integrative way, the PRQ is especially suited well to assess the subject-specific physiological or pathophysiological state in general. Such an assessment is much more being considered as relevant for modern physiological studies, which increasingly also focus on the individual physiology, as well as for a subject-specific medical diagnosis, disease monitoring, and treatment in the context of the paradigm of a “predictive, preventive, personalized, and participatory (P4)” medicine (Hood and Friend, 2011; Hood and Flores, 2012; Sagner et al., 2017). The PRQ also fits neatly into physiological and medical concepts that view human physiology and medicine in a more integrated and systemic way like the approaches of “systems biology” (Kitano, 2002; Somvanshi and Venkatesh, 2013), “integrative human physiology” (Coleman et al., 2011; Mackay et al., 2016), “functional medicine” (Jones and Quinn, 2010; Bland, 2015), “integrative medicine” (Barrett et al., 2003; Mcewen, 2017), and “personalized medicine” (Hamburg and Collins, 2010; Goetz and Schork, 2018).

The PRQ might be also a useful parameter to be included in multimodal neuroscientific studies that incorporate the measurement of systemic physiological variability alongside the changes in brain activity. While the measurement of HR and RR alongside detections of brain activity with functional magnetic resonance imaging (fMRI) has been conducted in numerous studies and is considered beneficial for a correct and integrated interpretation of fMRI data (Bulte and Wartolowska, 2017), this approach has only recently also been introduced for optical human neuroimaging studies with functional near-infrared spectroscopy (fNIRS). Our group pioneered this approach termed “systemic physiology augmented functional near-infrared spectroscopy” (SPA-fNIRS) (Metz et al., 2017; Scholkmann et al., 2017) and was the first to measure stimulus-evoked changes of the PRQ with simultaneous assessment of the changes in cerebral tissue oxygenation and blood perfusion (Scholkmann et al., 2017). To the best of our knowledge, we were also the first to investigate the relationship between absolute cerebral tissue oxygenation and rs-PRQ values (Scholkmann et al., 2019). The application of the PRQ in neuroscientific research is only just starting and harbors great potential for future insights into the coupling between neuronal activity and systemic physiological activity, and the cardiorespiratory system in particular.

Understanding human physiology and pathophysiology by analyzing the PRQ is a paradigm that should be studied in detail with new investigations and studies. According to our view, the following topics and research questions are of particular relevance for future studies:

- Definition of PRQ reference charts (for all types of PRQ parameters) depending, at the minimum, on sex and physiological constitution. New studies are needed to extend, e.g., the age range (preterm neonates, term neonates, infants, and elderly adults), and to create multi-dimensional reference charts, i.e., linking normal PRQ values with all these three factors simultaneously.
- Investigating the effect of factors hitherto not considered potential relevant for a proper interpretation of PRQ values, like gender (as opposed to sex) (Schiebinger and Stefanick, 2016) or race, ethnicity, and ancestry (REA) (Burchard et al., 2003; Popejoy et al., 2018). Also, investigating the links between individual PRQ behavior and genome-wide polygenic trait scores (PTS) associated with individuals’ phenotypes would be interesting (Chande et al., 2018).
- New studies are needed to investigate how the PRQ is linked with disease (healthy vs. disease subjects), disease progression, and therapy effects. There is also a need to research how the PRQ can be aligned to existing medical models, i.e., traditional, alternative, and complementary.
- It might be worth examining how the PRQ is linked with states of the other systems of human physiology (e.g., autonomic nervous system, central nervous system, immune system, and endocrine system) in order to gain novel insights into human physiology and pathophysiology.
- Ultradian and infradian changes of the PRQ should also be investigated in more detail. Of particular interest are infradian changes since there are already indications that the PRQ exhibits oscillations with weekly and monthly periods as discussed in section “The PRQ Is Time-Dependent: Ultradian, Circadian, and Infradian Rhythms.” It is likely that there is also a yearly rhythm. In addition, more detailed study is needed to explore how the PRQ is linked with environmental parameter changes (e.g., air temperature, air pressure, and air quality). Also, a link to cosmophysical phenomena should be taken into account since it has been shown already that the state of the CVS is influenced by heliogeophysical processes (Stoupel et al., 2002; Halberg et al., 2005; Pipin et al., 2010; Gadzhiev and Rakhmatulin, 2013; Zeng et al., 2014). Ultimately, the human *PRQ chronome* (i.e., the totality of all PRQ rhythms) should be investigated in detail, as was been done already, e.g., for the human blood pressure and heart rate chronomes (Ikonomov et al., 1991; Gubin et al., 1997).
- The circadian PRQ oscillation might be a particularly useful parameter to assess the overall physiological regulatory state of the human. For example, it would be interesting to investigate if there is a correlation between the subject-specific age-dependent circadian PRQ oscillation characteristics (magnitude and phase) and the activity state of the hypothalamic-pituitary-adrenal axis (HPA) determined by early life stress (ELS) experienced in childhood. That ELS determines the circadian cortisol oscillation in later life has been shown already (King et al., 2017). Studies relating to the individual circadian PRQ oscillation properties with psychological states and traits might also be of interest since it is already known that human chronobiology is linked to these psychological aspects of the human (Wirz-Justice, 2003; Monk et al., 2009; Taylor et al., 2011).

Besides these new studies needed in the field of human physiology and medical applications, there is also potential to extend the concept of the PRQ by developing methods that capture specific PRQ states or dynamics automatically. Such extensions have been used already in the previous studies. For example, novel ways of quantifying the PRQ distributions or capturing PRQ frequency locking time intervals have been developed and used already. There might also be the possibility to define *pulse-respiration rate variability* (PRQV), analog to the HRV; *pulse-respiration rate complexity* (PRQC), analog to the heart rate complexity (HRC) (Batchinsky et al., 2010; Brindle et al., 2016); or *pulse-respiration rate fragmentation* (PRQF), analog to the heart rate fragmentation (HRF) (Costa et al., 2017). PRQV, PRQC, and PRQF could then be quantified by the already existing methods developed for HRV, HRC, and HRF analyses.

Furthermore, since standard clinical patient monitors already measure the HR and RR, it would make sense to calculate and display the PRQ. This would automatically introduce the PRQ to clinicians, which could help spread the use of the parameter also for new studies and standard clinical monitoring. We encourage manufacturers of clinical patient monitors to update their device software to include the PRQ in the parameters determined and displayed. It is important that manufacturers and reaching hospitals also take measures to raise awareness of the new parameter. Since the PRQ is strongly modulated by chronobiology, using clinical monitors showing the PRQ might also encourage clinicians to take into account the chronobiological variability of physiological variables. As was pointed out recently by Mckenna et al. (2018), “critical care traditionally focuses on the ‘normalisation’ of physiological indices despite a limited evidence base (Holst et al., 2014; Gotts and Matthay, 2016), but preservation of circadian physiology is not part of clinical practice.” According to the authors, it is therefore possible that “neglecting the influence of circadian rhythmicity could contribute to the apparent lack of benefit from the majority of critical care targets tested in randomised controlled trials.”

In conclusion, our review article lists and summarizes key aspects of the PRQ and provides a detailed discussion of the PRQ’s role in future research about human physiology and pathophysiology, including in the medical context. We hope that we motivate the readers to use the PRQ in own studies and to participate in the further research about the PRQ in general. The time is ripe for re-discovery of the PRQ with respect to human physiology and medicine.

## Author Contributions

FS and UW contributed to the conception and design of the work and revised the first draft. FS conducted the literature research, organized the material, drafted the first version of the manuscript, both created the final version.

### Conflict of Interest Statement

The authors declare that the research was conducted in the absence of any commercial or financial relationships that could be construed as a potential conflict of interest.

## Footnotes

^1^To the best of our knowledge, the first time the parameter was termed “pulse-respiration quotient” was in the 1970s by the group of Hildebrandt. In the German literature, the parameter was named differently depending on the research group, or researcher, e.g., “Puls-Atem-Quotient” (PAQ), or “Quotient Puls Atem” (QPA or QP/A). Recently, the English terms “heart respiratory rate quotient” and “heart respiration ratio (HRR)” have been also used (Von Bonin et al., 2014).

^2^Also the term “physical constitution” is used in the literature. In our review paper, we use the term “physiological constitution” as an umbrella term for both physiological and anatomical aspects, without including psychological ones.
